# Toxin-Induced Necroptosis Is a Major Mechanism of *Staphylococcus aureus* Lung Damage

**DOI:** 10.1371/journal.ppat.1004820

**Published:** 2015-04-16

**Authors:** Kipyegon Kitur, Dane Parker, Pamela Nieto, Danielle S. Ahn, Taylor S. Cohen, Samuel Chung, Sarah Wachtel, Susan Bueno, Alice Prince

**Affiliations:** 1 Department of Pharmacology, Columbia University Graduate School of Arts and Sciences, Columbia University, New York, New York, United States of America; 2 Department of Pediatrics, College of Physicians and Surgeons, Columbia University, New York, New York, United States of America; 3 Department of Molecular Genetics and Microbiology, Pontifical Catholic University of Chile, Santiago, Chile; Johns Hopkins School of Medicine, UNITED STATES

## Abstract

*Staphylococcus aureus* USA300 strains cause a highly inflammatory necrotizing pneumonia. The virulence of this strain has been attributed to its expression of multiple toxins that have diverse targets including ADAM10, NLRP3 and CD11b. We demonstrate that induction of necroptosis through RIP1/RIP3/MLKL signaling is a major consequence of *S*. *aureus* toxin production. Cytotoxicity could be prevented by inhibiting either RIP1 or MLKL signaling and *S*. *aureus* mutants lacking *agr*, *hla* or Hla pore formation, *lukAB* or *psms* were deficient in inducing cell death in human and murine immune cells. Toxin-associated pore formation was essential, as cell death was blocked by exogenous K+ or dextran. MLKL inhibition also blocked caspase-1 and IL-1β production, suggesting a link to the inflammasome. *Rip3* *^-/-^* mice exhibited significantly improved staphylococcal clearance and retained an alveolar macrophage population with CD200R and CD206 markers in the setting of acute infection, suggesting increased susceptibility of these leukocytes to necroptosis. The importance of this anti-inflammatory signaling was indicated by the correlation between improved outcome and significantly decreased expression of KC, IL-6, TNF, IL-1α and IL-1β in infected mice. These findings indicate that toxin-induced necroptosis is a major cause of lung pathology in *S*. *aureus* pneumonia and suggest the possibility of targeting components of this signaling pathway as a therapeutic strategy.

## Author Summary

*Staphylococcus aureus* (SA) cause a highly inflammatory pneumonia associated with substantial morbidity and mortality. Much of this lung destruction is attributed to toxins that target specific receptors on human and murine cells. We demonstrate that the α-hemolysin (Hla) and other *agr*-regulated toxins activate RIP1/RIP3/MLKL-mediated necroptosis and IL-1β expression, through a mechanism that involves MLKL pore-formation and inflammasome activation. Cell death can be inhibited by osmoprotectants and K^+^ repletion. Necroptosis results in alveolar macrophage depletion and loss of anti-inflammatory signaling. *Rip3* ^*-/-*^ mice maintain significantly greater numbers of alveolar macrophages with anti-inflammatory phenotypes, CD206^+^ and CD200R^+^; decreased proinflammatory cytokine production; and improved SA clearance. Necroptosis represents a common mechanism of pulmonary damage activated by multiple SA toxins.

## Introduction

*Staphylococcus aureus* (SA) is a major cause of pneumonia in healthcare associated settings and especially as a complication of influenza [1, 2]. The methicillin resistant *S*. *aureus* (MRSA) USA300 strain that is currently epidemic in the United States is especially virulent, associated with substantial morbidity and mortality [3]. Much of the pulmonary pathology associated with *S*. *aureus* pneumonia has been attributed to its collection of virulence factors that include genes facilitating colonization, a major factor in increasing susceptibility to subsequent invasive infection [4], in combination with expression of multiple toxins. These toxins include, α-hemolysin (Hla), which recognizes ADAM10 in the lung [5] and activates the NLRP3 inflammasome [6] and multiple leukotoxins such as LukAB [7, 8] and the PSMs [9]. Toxin production causes pulmonary damage demonstrated in murine models [5, 10] and confirmed using human tissue, as receptors for several of these toxins are species specific [11].

Hla, for example, targets ADAM10 and *Adam10* ^*-/-*^ mice are protected from SA pneumonia with significantly decreased pathology and systemic dissemination [5]. Hla also activates the NLRP3 inflammasome stimulating production of IL-1β and IL-18, highly proinflammatory cytokines important for neutrophil recruitment and critical for staphylococcal eradication [6]. However, *Nlrp3* ^*-/-*^ mice did not have a major phenotype in the setting of SA pneumonia [10], suggesting that inflammasome activation by itself may not be critical in eliciting host damage. In other models of SA pneumonia the intensity of the host response, excessive local inflammation and tissue damage impede bacterial clearance. Models of staphylococcal pneumonia in mice lacking components of innate immune signaling, such as the type I IFN receptor null (*Ifnar* ^*-/-*^) mice [12, 13], tumor necrosis factor receptor 1 null (*Tnfr1* ^*-/-*^) mice [14], type III interferon (IFNλ) receptor null (*Il28r* ^*-/-*^) mice [15] or nucleotide-binding oligomerization domain-containing protein 2 null (*Nod2* ^*-/-*^) mice [16], have significantly improved outcomes. Given the multiple cell types in the lung that participate in proinflammatory signaling and the numerous mechanisms through which SA target these recruited immune cells, we postulated that the specific mode(s) of cell death induced by SA contribute to lung pathology.

Bacteria stimulate distinct mechanisms of cell death, some of which are highly proinflammatory, such as pyroptosis and necroptosis. Toxin producing bacteria including *S*. *aureus* activate pyroptosis, a caspase-1 dependent form of cell death that generates IL-1β and IL-18 and the inflammatory responses associated with these cytokines [17]. Apoptosis or autophagy may also contribute to pathogen clearance but do not elicit host inflammation. Necroptosis is a highly proinflammatory mechanism of cell death [18]. In the absence of caspase-8 activation, receptor-interacting serine-threonine kinase (RIP)1 and RIP3 interacts through the RIP homotypic interaction motifs (RHIMs) activating RIP3, which in turn phosphorylates mixed lineage kinase domain-like (MLKL) leading to MLKL pore formation and loss of plasma membrane integrity [18–22]. The toxicity associated with several major bacterial pathogens such as *Salmonella enterica* serovar typhimurium and *Yersinia pestis* has been ascribed to the induction of necroptosis [18, 21, 23, 24]. While necroptosis associated cytokine expression serves to recruit phagocytes and eliminate infected cells, it also results in substantial tissue damage and cell loss. Thus, in the process of pathogen clearance, specific host responses can contribute to damaging inflammation resulting in significant morbidity and mortality.

In the lung particularly, where inflammation interferes with normal physiology and gas exchange, pro-inflammatory modes of cell death may increase pathology. Moreover, the loss of specific types of immune cells, which are not immediately replenished in the lung, may affect the host’s ability to regulate inflammation. In the experiments detailed in this report, we demonstrate that SA toxins induce necroptosis, which is responsible for much of the inflammatory pathology characteristic of SA pneumonia.

## Results

### SA induces necroptosis in mouse and human macrophages

**Fig 1:**
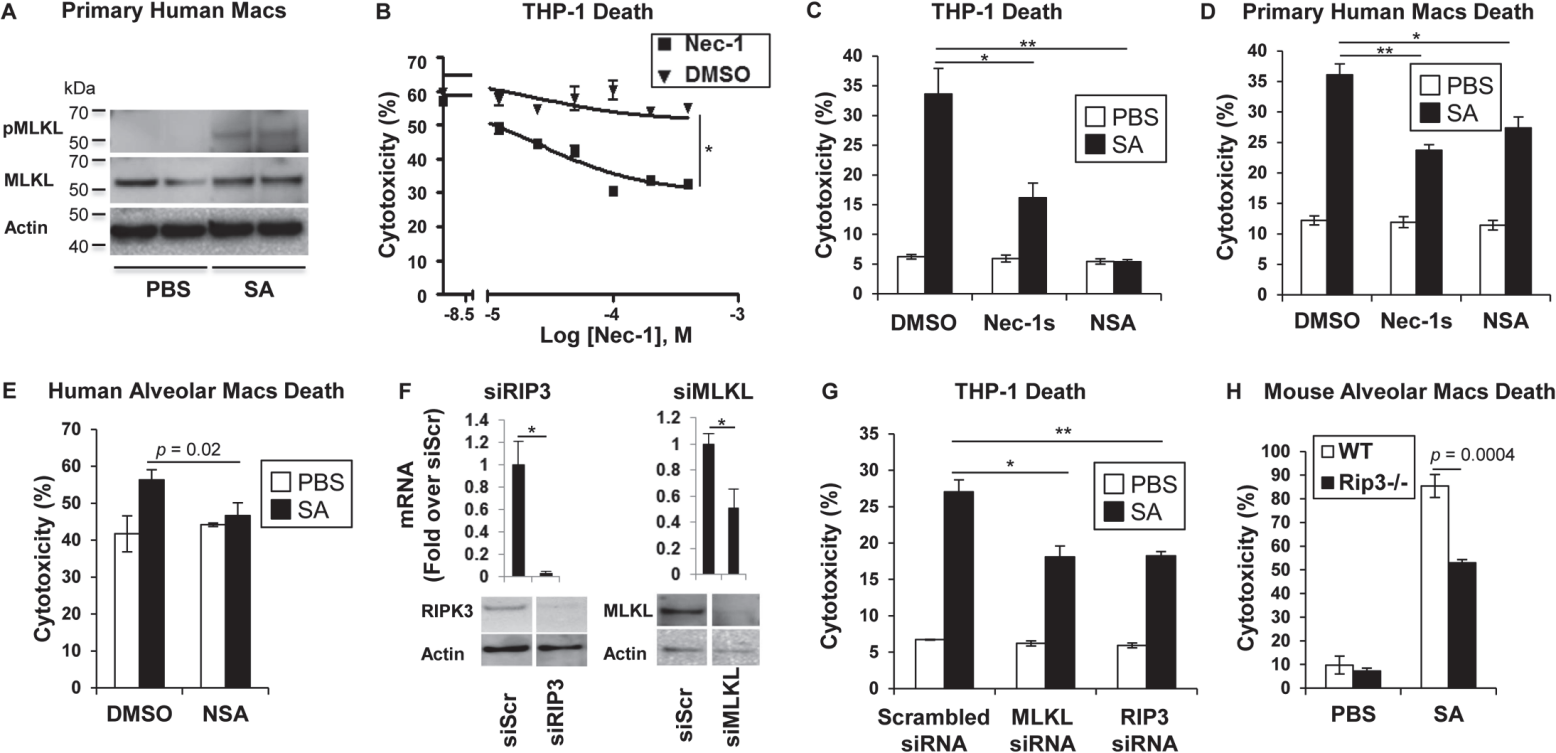
SA induces necroptosis in macrophages. (**A**) Primary human macrophages were stimulated for 2 hours with SA MOI10 or PBS and probed with antibodies against phosphorylated MLKL (pMLKL), MLKL and actin. (**B**) Cytotoxicity in THP-1 cells pretreated for 1 hour with necrostatin-1 (Nec-1) or DMSO and incubated with SA MOI10 for 2 hours determined by LDH assay (inhibitory concentration [IC50], *p < 0.0001). (**C**) Cytotoxicity in THP-1 cells pretreated for 1 hour with 200 μM necrostatin-1 stable (Nec-1s), 10 μM necrosulfonamide (NSA) or DMSO (*p = 0.0037, ** *p* = 0.0004) and stimulated with SA MOI10 for 2 hours. (**D**) Cytotoxicity in primary human macrophages pretreated with 10 μM NSA, 200 μM Nec-1s or DMSO (*p = 0.04, **p = 0.0004) and stimulated with SA MOI10 for 2 hours. (**E**) Cytotoxicity in freshly obtained human alveolar macrophages pretreated with 10 μM necrosulfonamide (NSA) or DMSO) and stimulated with SA MOI100 for 2 hours. (**F**) THP-1 cells were transfected for 72 hours with RIP3, MLKL small interfering RNA (siRNA) or siRNA scrambled control (siScr). mRNA and protein levels of RIP3 and MLKL was measured by quantitative real time PCR and western blot after (*p < 0.05). (**G**) Cytotoxicity in THP-1 cells with depleted RIP3 or MLKL and infected with SA MOI10 for 2 hours. (*p = 0.0022, **p = 0.001). (**H**) Cytotoxicity in primary alveolar macrophages from WT and *Rip3-/-* mice incubated with USA300 MOI100 for 2 hours. Data are representative of two independent experiments with three technical replicates (**B-H,** mean and SD) or two technical replicates (**A**). *p* values were determined by one-way ANOVA followed by Bonferroni Corrections to correct for multiple comparisons (**C, D, G**) or two-tailed Student's *t* test (**B, E, F, H**).

MRSA USA300 pneumonia is associated with toxin production causing an especially damaging, necrotizing pneumonia. We postulated that RIP1/RIP3/MLKL-mediated necroptosis was likely to be involved in SA-induced cytotoxicity. Direct evidence of the participation of MLKL in this pathway was obtained by documenting SA-induced phosphorylation of MLKL in primary human macrophages (**Fig 1A**). Biochemical inhibitors were then used to determine the importance of this pathway in human and murine immune cells. In the presence of necrostatin-1 (Nec-1), which targets RIP1 [25], a dose-dependent inhibition of USA300 induced cytotoxicity was observed in THP-1 cells, a human monocyte-macrophage cell line (**Fig 1B**). Both Nec-1s (a specific and stable analog of Nec-1) [26] and necrosulfonamide (NSA), a specific and potent inhibitor of MLKL [27], protected THP-1 cells and primary human macrophages from SA induced cytotoxicity (**Fig 1C and 1D**). MLKL inhibition by NSA also protected freshly harvested human airway macrophages from SA-induced cytotoxicity (**Fig 1E**); as did gene silencing using siRNA to target *RIP3* or *MLKL* in the THP -1 cells (**Fig 1F and 1G**).

Although several staphylococcal toxins have specificity for human receptors [11], the murine model of pneumonia replicates much of the pathology observed in human infection [5], suggesting murine susceptibility to at least some of the same toxicities. Freshly harvested murine pulmonary macrophages from *Rip3* ^*-/-*^ mice were found to be significantly protected from cell death when compared to wild type (WT) macrophage controls exposed to SA (**Fig 1H),** suggesting that necroptosis is also activated in murine immune cells.

### Secreted SA toxins are sufficient to induce cell death

**Fig 2:**
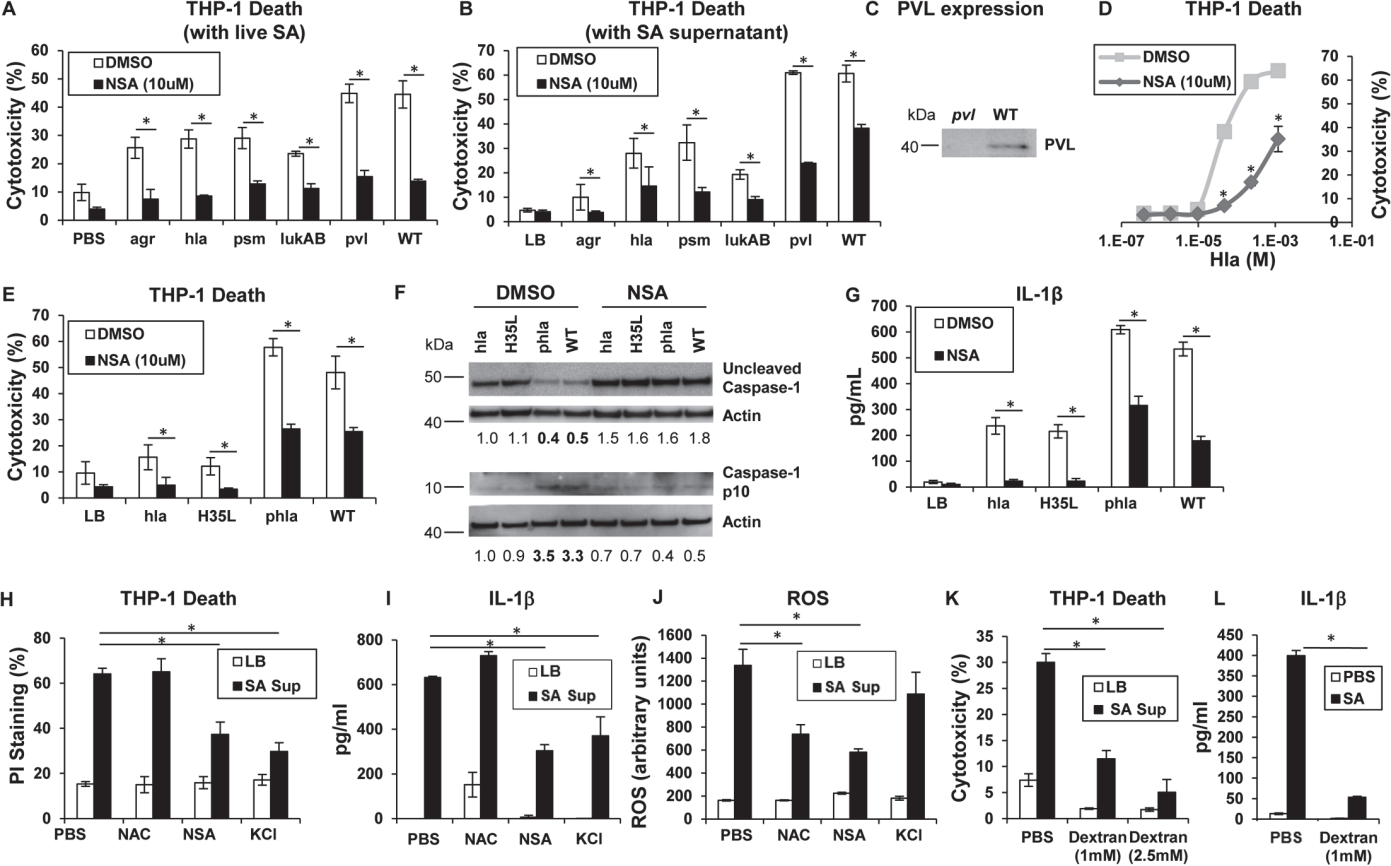
Necroptosis and inflammasome activation share signaling components (**A**) Cytotoxicity in THP-1 cells treated with MRSA USA300 (WT), *agr* null, *hla* null, *psm*(*α/β/hld*) null, *lukAB* null or *pvl* (*lukSF*) null mutants for 2 hours (*p < 0.005). (**B**) Cytotoxicity in THP-1 cells pretreated with 10 μM NSA or DMSO and exposed to supernatant harvested from MRSA USA300 (WT), *agr*, *hla*, *psm* (Δ*α/β/hld*), *lukAB* and *pvl* mutants (*p < 0.0001). (**C**) PVL expression in supernatant from *pvl* and WT SA as assayed by western blot. (**D**) Cytotoxicity in THP-1 cells pretreated with 10 μM NSA or DMSO and exposed to increasing concentrations of purified Hla for 2 hours (*p < 0.05). (**E**) Cytotoxicity in THP-1 cells pretreated with 10 μM NSA or DMSO and exposed to supernatant harvested from *hla* mutant, *hla* mutant complemented with *hla*H35L or *hla* plasmid and WT (*p < 0.05). (**F**) Caspase-1 western blot of THP-1 cells pretreated with 10 μM NSA or DMSO and exposed to SA supernatant for 2 hours. Levels of uncleaved caspase-1 and caspase-1 p10 fragment over actin as determined by densitometry are shown. (**G**) Levels of IL-1β released by THP-1 cells pretreated with 10 μM NSA or DMSO and exposed to SA supernatant for 2 hours quantified by ELISA (*p < 0.05). (**H**) Cytotoxicity as measured by PI staining, (**I**) IL-1β and (**J**) ROS in THP-1 cells pretreated with 1 mM N-acetylcysteine (NAC), 10 μM NSA, 60 mM KCl or PBS and exposed to SA supernatant for 2 hours (*p < 0.005). (**K**) Cytotoxicity as measured by LDH assay in THP-1 cells pretreated with 1 mM, 2.5 mM dextran or PBS and exposed to SA supernatant for 2 hours. (**L**) IL-1β in samples in (**K**). Data are representative of two independent experiments with three technical replicates (mean and SD). *p* values were determined by one-way ANOVA followed by Bonferroni Corrections to correct for multiple comparisons (**H, I, J,** K) or two-tailed Student's *t* test (**A, B, E, G, L**).

Phagocytosis of intact staphylococci and the effect of isolated toxins are associated with cytotoxicity. Several SA mutants were compared to determine if a specific toxin was primarily associated with cytolysis of human cells, if secreted products are sufficient to cause cell death and if toxicity could be prevented with a MLKL inhibitor. An *agr* null mutant and mutants in discrete toxin genes were studied. The *agr* locus is a central regulator of several important toxins including α-hemolysin (Hla), a major factor in lung injury [5], PSMs [9] and expression of the two-component toxin LukA/B [28]. NSA significantly inhibited macrophage cytotoxicity induced by infection with *agr* (although overall toxicity associated with this mutant was low), *hla*, *psm* (*psmα/β/hld*), *lukAB*, *lukSF* (*pvl*) and WT MRSA USA300 (**Fig 2A**). Culture supernatants harvested from the *agr* mutant did not confer significant cytotoxicity, nor were supernatants from *hla* null, *psm* null or *lukAB* null mutants significantly cytotoxic (**Fig 2B**). *Pvl* did not appear to play a significant role in THP-1 death under our experimental conditions (**Fig 2A and 2B**), despite demonstrable PVL production (**Fig 2C**). Cytotoxicity associated with WT supernatant was significantly, but not completely inhibited by NSA (**Fig 2B**). Although these culture supernatants contain shed pathogen-associated molecular patterns (PAMPs) as well as toxins, these studies indicate that multiple secreted staphylococcal products in the absence of intact bacteria or phagocytosis induce cell death.

### Hla pore formation activates necroptosis and shared elements of the inflammasome

Hla is a major *agr*-dependent staphylococcal toxin that activates NLRP3 inflammasome signaling through pore formation and K^+^ loss resulting in both cell death and IL-1β expression [6, 29]. Purified Hla, when added to THP-1 cells, induced a dose-dependent cytotoxicity that was significantly decreased by NSA (**Fig 2D**). We observed that Hla-dependent pore formation stimulates necroptosis in THP-1 cells and a Hla mutant H35L, lacking a functional pore [5] induced minimal cytotoxicity in contrast to the WT or a complemented *hla* null mutant (phla) (Figs **2E and S1A**). Hla pore formation is required for NLRP3 inflammasome activation and we confirmed that Hla was unable to activate caspase-1, as monitored by the generation of the p10 caspase-1 cleavage product (**Fig 2F**). Since MLKL pore formation is a central component of necroptosis, we used NSA to determine if MLKL is also involved in inflammasome activation. In the presence of NSA, caspase-1 activation (**Fig 2F**) and generation of IL-1β, a product of the NLRP3 inflammasome, were both inhibited (**Fig 2G**). Inhibition of MLKL also significantly decreased the amount of IL-1β stimulated by purified Hla (**S1B Fig**). These results suggest that MLKL pore formation is associated with inflammasome activity, an observation consistent with studies linking the inflammasome and the necrosome components RIP1/RIP3 and MLKL [30–32].

The association of MLKL pore formation and stimuli that lead to activation of the inflammasome was further demonstrated with prevention of cell death as well as the generation of IL-1β by addition of exogenous K^+^ (**Fig 2H and 2I**). However, generation of ROS, which is often cited as an inflammasome agonist [33], was not involved, as N-acetyl-cysteine (NAC) did not inhibit either cytotoxicity or IL-1β generated by SA supernatant (**Fig 2H, 2I and 2J**). Dextran, which acts as an osmoprotectant [34], protected THP-1 cells from SA induced cytotoxicity and significantly inhibited IL-1β production (**Fig 2K and 2L**), consistent with effects on both necroptosis and inflammasome activation. As the MLKL inhibitor NSA prevented inflammasome-associated responses, namely caspase-1 cleavage and IL-1β generation, it appears that necrosome components contribute to activation of the inflammasome.

### Inhibition of necroptosis improves SA clearance in vivo

**Fig 3:**
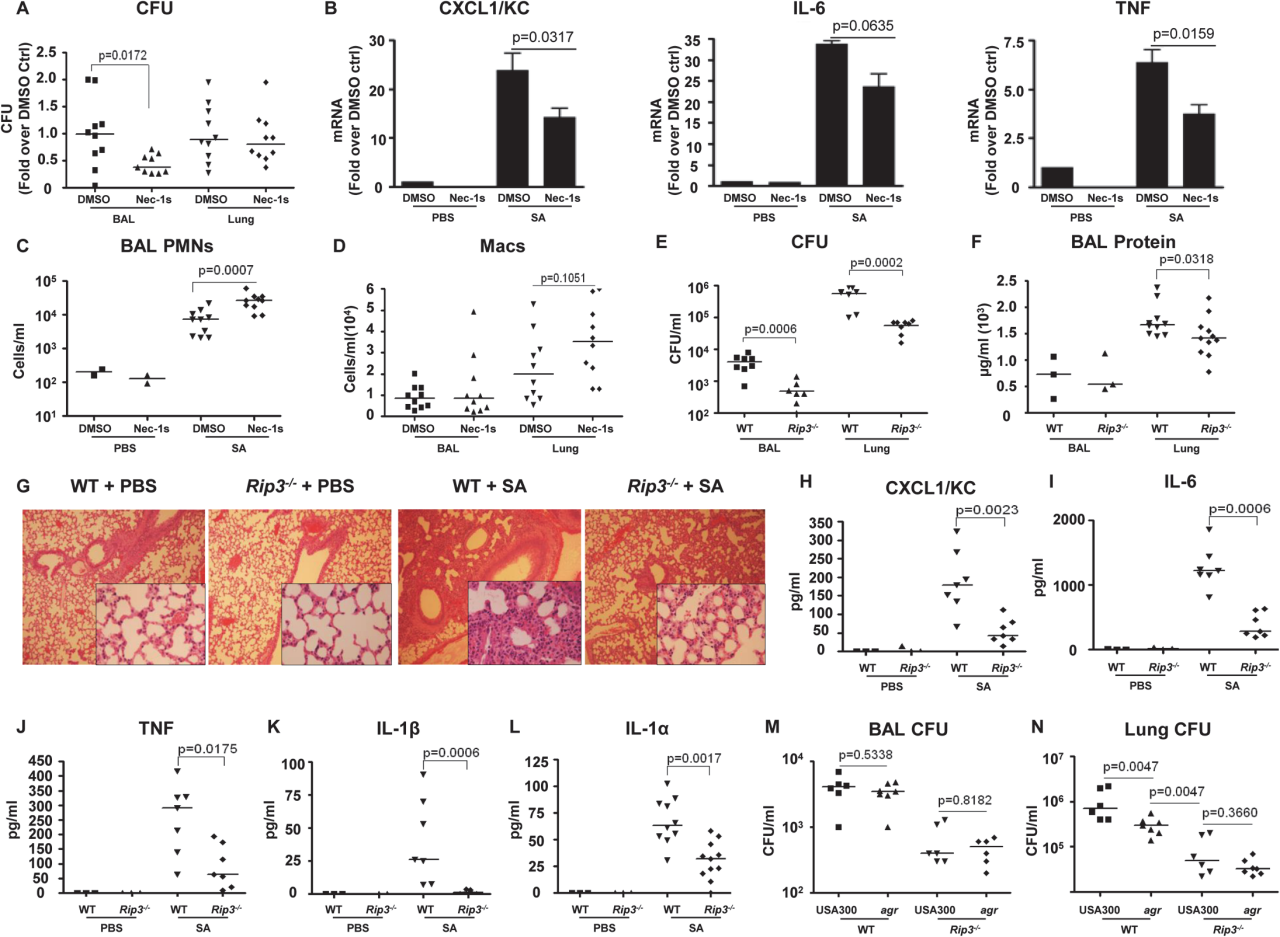
Blockade of necroptosis improves bacterial clearance. (**A**) C57BL/6J mice were treated with necrostatin-1 stable (Nec-1s) or DMSO and infected with MRSA USA300 (SA). CFU recovered in BAL and lung were quantified and normalized to DMSO controls (n = 2 for PBS and n = 10 per SA group). (**B**) *Cxcl1/Kc*, *Il-6* and *Tnf* expression in the lung homogenate quantified by real time PCR compared to control (ctrl). (**C,D**) Neutrophils (PMNs) in BAL and macrophages (Macs) in BAL and lung. (**E**) *Rip3-/-* or WT mice were infected with MRSA USA300 (SA) for 18 hours and CFU recovered in BAL and lung quantified (n = 3 for PBS and n = 8 per SA group). (**F**) Protein in the BAL fluid of WT and *Rip3-/-* mice. (**G**) Hematoxylin and eosin stain (H&E) staining of WT and *Rip3-/-* mice lungs (magnification of 100x; insert, magnification of 400x). (**H-L**) CXCL1/KC, IL-6, TNF, IL-1β and IL-1α levels in the BAL fluid measured by ELISA. (**M, N**) *Rip3-/-* or WT mice were infected with *agr* null or MRSA USA300 (USA300) for 18 hours and CFU recovered in BAL and lung quantified (n = 6 per group).Gating strategies for immune cells are in **S2E Fig.** Data are pooled from two independent experiments. Each point represents a mouse. Lines show median values. *p* values were determined by nonparametric Mann-Whitney test.

To determine the importance of SA-induced necroptosis in the pathogenesis of SA pneumonia, we treated mice with necrostatin-1s (Nec-1s), a stable, specific and potent analog of necrostatin-1[26], at 18 hours prior to and at the time of SA intranasal inoculation. The Nec-1s-treated mice, as compared with PBS treated controls, had significantly improved clearance of bacteria from the BAL fluid at 4 hours post inoculation (p = 0.0172) (**Fig 3A**). Nec-1s treatment resulted in decreased transcription of KC (p = 0.0317), IL-6 (p = 0.0635) and TNF (p = 0.0159) in the lung (**Fig 3B**). However, protein levels of the cytokines in the BAL were not altered (**S2A Fig**), likely due to the short exposure to the inhibitor. Nonetheless, the numbers of neutrophils (PMNs) in the Nec-1s-treated mice were increased in the BAL, suggesting that inhibiting necroptosis could preserve neutrophil viability (**Fig 3C**). There was also a trend toward increased numbers of macrophages (Macs) (**Fig 3D**), but Nec-1s treatment did not affect the abundance of other immune cells (**S2B Fig**).

The involvement of RIP3 was then evaluated by comparing the response of wild type C57BL/6J (WT) versus *Rip3* ^*-/-*^ mice to SA infection. At 24 hours post inoculation, there was significantly improved USA300 clearance from both the bronchoalveolar lavage fluid (BAL) (p = 0.0006) and lung (p = 0.0002) of the *Rip3* ^*-/-*^ mice as compared with WT mice (**Fig 3E**). Barrier function, as measured by protein content in BAL, was less disrupted (**Fig 3F**), and lung architecture was better preserved (**Fig 3G**) in the *Rip3* ^*-/-*^ mice compared with the controls. As observed with the pharmacological inhibition of necroptosis, the *Rip3* ^*-/-*^ mice had significantly less induction of proinflammatory cytokine/chemokine expression, KC, IL-6, TNF, IL-1α and IL-1β (**Fig 3H–3L**). IL-1β, which is associated with NLRP3 activation [6], was strikingly low in *Rip3* ^*-/-*^ mice (**Fig 3K**), consistent with a potential role of RIP3 in NLRP3 inflammasome regulation. The mRNA levels of KC and IL-6, but not TNF, were also decreased in the lungs of *Rip3* ^*-/-*^ mice compared to WT controls (**S2C Fig**), suggesting that decreased inflammatory signaling is associated with improved staphylococcal clearance.

The activation of RIP3-mediated signaling by SA toxins was further confirmed by monitoring the outcome of WT versus *Rip3* ^*-/-*^ mice infected with *agr* null USA300. Although WT mice were able to clear *agr* null USA300 more efficiently than WT USA300 from the lung, the *Rip3* ^*-/-*^ mice cleared *agr* and WT SA strains equally well (**Fig 3M and 3N**). We noted that the *agr* mutant was cleared significantly more from the lungs of the *Rip3-/-* than from the WT mice (**Fig 3N**), indicating that *agr*-independent gene products are likely to contribute to necroptosis as well. Unlike WT USA300, *agr* null mutants did not cause loss of lung architecture in *Rip3* ^*-/-*^ and WT mice (**S2D Fig**). These results are consistent with our hypothesis that *agr* dependent toxins activate necroptosis contributing to virulence, but suggest multiple gene products contribute to this inflammatory form of cell death.

### Loss of macrophage populations by necroptosis

**Fig 4:**
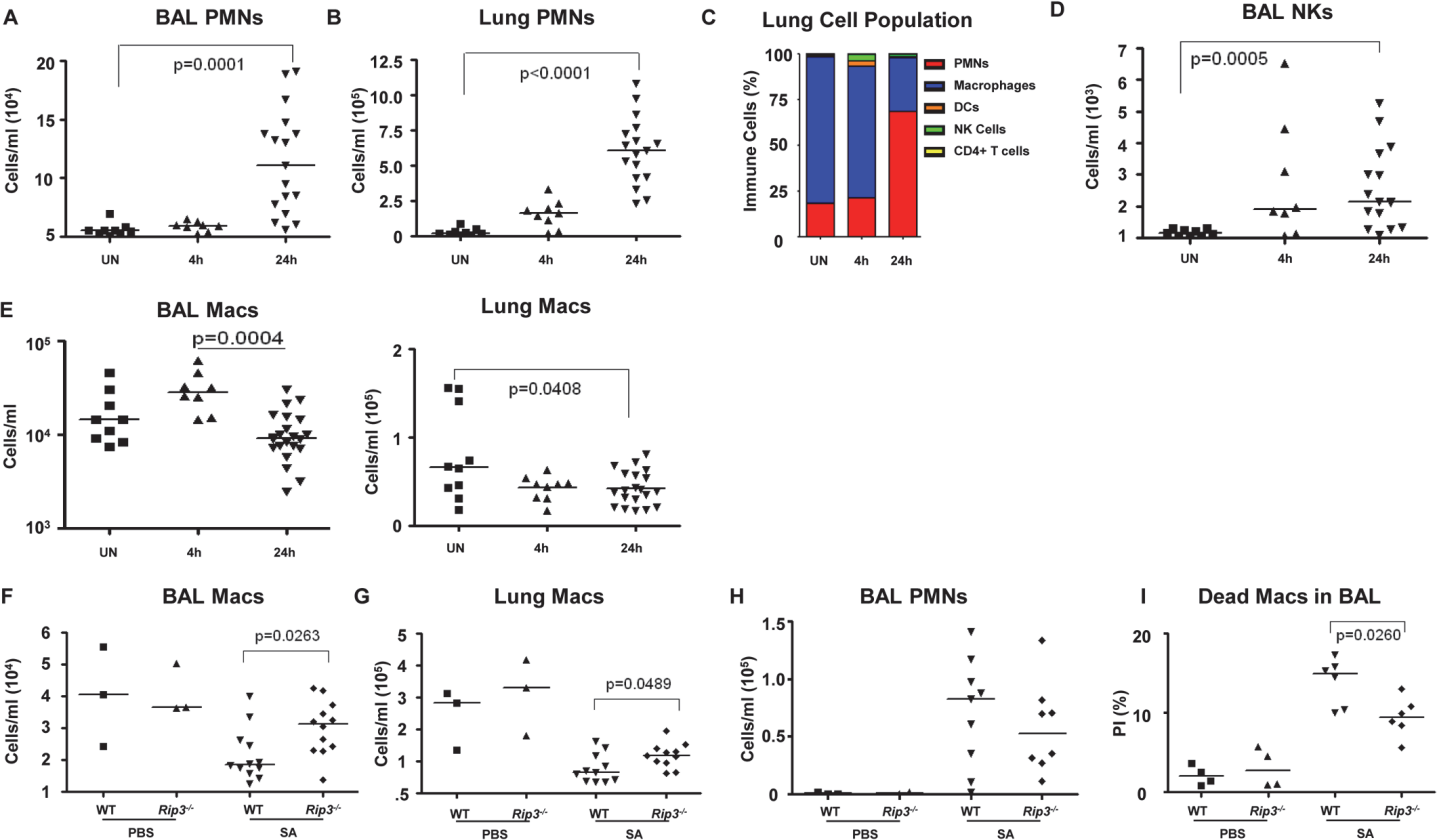
Macrophage expansion is limited during SA pneumonia. (**A-E**) Mice were infected intranasally with 10^7^ CFU/mouse MRSA USA300 and their lung homogenate analyzed by FACS for immune cell populations at 4 and 24 hours as compared to uninfected controls (UN) (n = 9 for UN, n = 9 for 4 h and n = 20 for 24 h for SA groups). (**A, B**) PMNs in BAL and lung quantified. (**C**) Percentages of immune cell populations at 4 and 24 hours as compared to uninfected controls (UN) were determined. (**D**) Natural killer cells (NKs) in BAL. (**E**) Macrophages in BAL and lung. (**F-I**) *Rip3-/-* and WT mice infected with SA for 18 hours (n = 3 for PBS and n = 8 per SA group). (**F, G**) Macrophages in BAL and lung of *Rip3-/-* and WT mice. (**H**) PMNs in BAL of *Rip3-/-* and WT mice. (**I**) Propidium iodide positive (PI^+^) macrophages in the BAL of WT and *Rip3-/-* mice. Data are pooled from three independent experiments. Each point in represents a mouse. Lines show median values. *p* values are indicated for significantly different comparisons (nonparametric Mann-Whitney test).

The loss of specific populations of immune cells through necroptosis is likely to influence the outcome of infection, particularly the loss of resident cells in the lung that may not be immediately replenished. We used flow cytometry to quantify the relative and absolute numbers of immune cells in both bronchoalveolar lavage fluid as well as in the lung itself over the initial stages of acute SA pulmonary infection (**Fig 4**). In WT mice, immune cell recruitment was dominated by neutrophils with 10-fold and 20-fold increases in both the BAL and lung respectively at 24 hours post-infection (**Fig 4A and 4B**) and an increase in overall percentage compared to other immune cells (**Fig 4C**). Natural killer cells (NKs), dendritic cells (DCs) and CD4^+^ T cells were also readily detected after infection, with increases in NKs noted in the BAL (Figs **4D and S3A–S3D**). The number of macrophages in the BAL increased at 4 hours post-intranasal SA inoculation, but decreased significantly in the lung and BAL 24 hours post-infection (**Fig 4E**), despite the production of proinflammatory cytokines and chemokines, consistent with ongoing loss in the setting of SA infection. Much of this macrophage loss could be attributed to necroptosis as in comparison with the WT mice the *Rip3* ^*-/-*^ mice had significantly increased numbers of macrophages in both BAL and lung (**Fig 4F and 4G**), but equivalent numbers of neutrophils in BAL (**Fig 4H**). To better assess macrophage viability, we quantified the number of dead macrophages in mouse BAL (PI^+^, CD11c^+^MHCII^low^) at 24 hours post-inoculation. *Rip3* ^*-/-*^ mice had fewer PI^+^ macrophages compared to WT controls (Figs **4I and S3E**), but no significant differences in the numbers of other immune cell types including PMNs, CD4^+^ T cells, DCs and NK cells (**S3F–S3H Fig**).

**Fig 5:**
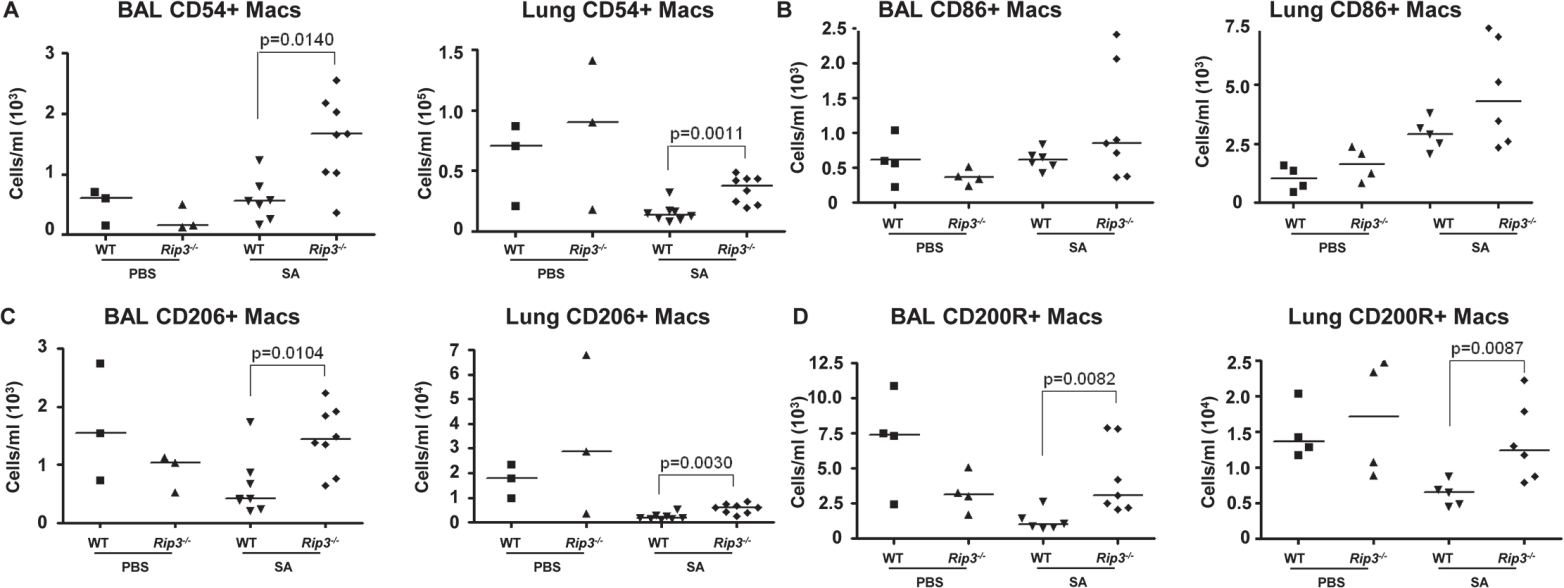
Pulmonary macrophages from *Rip3-/-* mice have an anti-inflammatory phenotype. (**A-C**) *Rip3-/-* or WT mice were infected with SA for 18 hours. (**A**) The absolute numbers of CD54^+^ macrophages in BAL and lung quantified (n = 3 for PBS, n = 8 for SA groups). (**B**) CD86^+^ macrophages in BAL and lung (n = 4 for PBS, n = 6 for WT with SA, n = 7 for *Rip3-/-* with SA). (**C**) CD206^+^ macrophages in BAL and lung (n = 3 for PBS, n = 7 for WT with SA, n = 8 for *Rip3-/-* with SA). (**D**) CD200 receptor positive (CD200R^+^) macrophages in BAL and lung (n = 3 for PBS, n = 7 for WT with SA, n = 8 for *Rip3-/-* with SA). Gating strategies for macrophage markers are in **S3E Fig.** Data are pooled from two independent experiments. Each point represents a mouse. Lines show median values. *p* values were determined by nonparametric Mann-Whitney test.

We next addressed the likely function of these alveolar macrophages in the immediate host response to SA. The *Rip3* ^*-/-*^ mice had a diminished bacterial burden (**Fig 3E**), fewer neutrophils (**Fig 4H**), perhaps because of decreased levels of infection, but increased numbers of macrophages in BAL and lung. Neutrophils are much more avidly phagocytic than macrophages [35], thus their diminished numbers may not impair their total phagocytic clearance. Resident alveolar macrophages at rest are generally skewed toward an anti-inflammatory phenotype with surface expression of CD206 and CD200R (M2 markers) and must be activated to the M1-like inflammatory phenotype in the context of an infection [36]. Macrophages from infected BAL of WT and *Rip3* ^*-/-*^ mice had significant upregulation of CD54 (ICAM-1), a major activation marker of macrophages, consistent with an immunostimulatory phenotype evoked by infection, shown in absolute numbers (**Fig 5A**) and as a percentage (**S4A Fig**). Both WT and *Rip3* ^*-/-*^ mice also displayed equivalent expression of the activation marker CD86 (Figs **5B and S4B**). CD206 and CD200 receptor (CD200R) [36, 37], both major anti-inflammatory receptors were significantly increased in macrophages recovered from *Rip3* ^*-/-*^ BAL and lung (Figs **5C, 5D, S4C, and S4D**). The corresponding increases in these regulatory markers on *Rip3* ^*-/-*^ pulmonary macrophages correlated with the decreased inflammatory response observed in the *Rip3* ^*-/-*^ mice compared to WT controls. These results suggested that macrophages are critical in controlling inflammation in the setting of acute SA infection and their loss through necroptosis contributes to the inflammation observed in the lung.

### Pulmonary macrophages are critical for SA clearance from the lung

**Fig 6:**
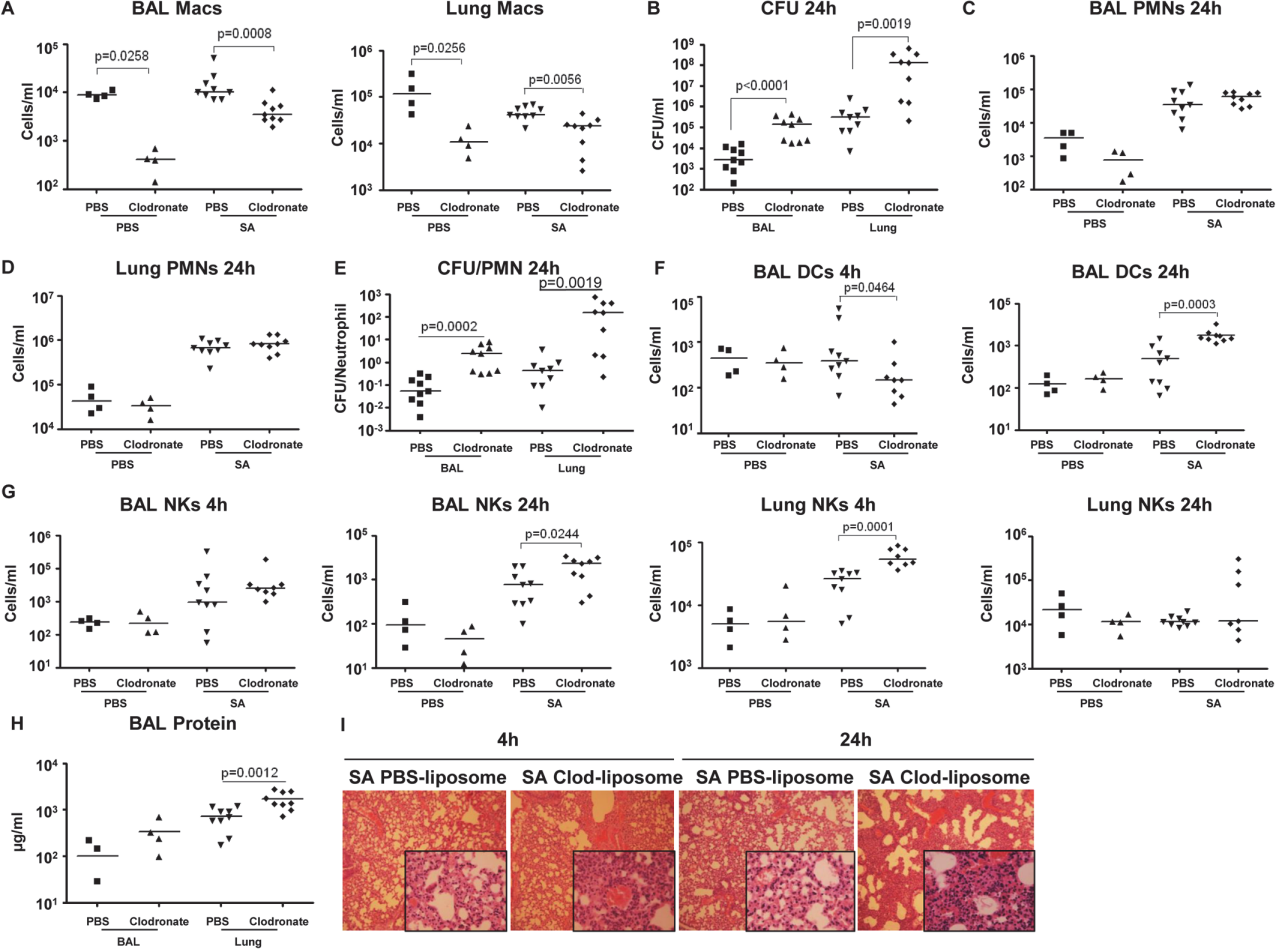
Depletion of pulmonary macrophages contributes to poor outcome. (**A**) C57BL/6J mice were treated with clodronate- or PBS-loaded liposomes for 24 hours. Mice were infected intranasally with SA and numbers of macrophages in BAL and lung quantified 24 hours post-infection by flow cytometry (n = 4 for PBS groups, n = 10 for SA groups). (**B**) Numbers of SA (CFU) recovered from BAL and lung were quantified at 4 and 24 hours post-infection. (**C, D**) PMNs in BAL and lung 24 hours post-infection. (**E**) Ratios of CFU to PMNs in BAL and lung. (**F**) DCs in BAL and lung 24 post-infection. (**G**) NKs in BAL and lung 4 hours and 24 hours post-infection. (**H**) Protein in the BAL fluid 24 hours post-infection. (**I**) Hematoxylin and eosin stain (H&E) staining of mouse lung (magnification 100x; insert, magnification 400x). FACS blots showing depleted macrophages and unchanged PMNs after clodronate treatment are in **S4D Fig.** Data are pooled from three independent experiments. Each point in represents a mouse. Lines show median values. *p* values obtained by nonparametric Mann-Whitney test.

To establish the importance of the resident alveolar macrophage population, which would be lost through toxin-induced necroptosis, we used clodronate-loaded liposomes to deplete airway macrophages, characterized SA clearance as previously described [38] and studied immune response in detail. The mice treated with clodronate, as compared with those treated with PBS-loaded liposomes had 95.4% (p = 0.0258) and 91.6% (p = 0.0256) decreases in macrophages recovered from BAL and lung respectively (**Fig 6A**). Macrophage depletion was accompanied by significantly increased staphylococcal load recovered from both BAL (p<0.0001) and lungs (p = 0.0019) of the clodronate as compared with the PBS treated mice (**Fig 6B**), but did not affect recruitment of neutrophils to the airway or lung (**Fig 6C and 6D**). However, there was a significantly greater ratio of staphylococci to neutrophils in the macrophage-depleted mice at 24 hours post infection (**Fig 6E**), suggesting that despite equivalent numbers of neutrophils, mice lacking macrophages were less able to control infection. While clodronate treatment resulted in decreased DC numbers in the BAL at 4 hours post infection, by 24 hours they were significantly increased in the clodronate treated group (Figs **6F and S5A**). There was an increase in NK cells in the lung by 4 hours post infection and in the BAL at 24 hours in the clodronate treated mice (**Fig 6G**). The CD4^+^ T cell population was not affected by macrophage depletion (**S5B Fig**). The net effect of this immune response is reflected in the protein content of BAL and the histopathology of the infected murine lungs demonstrating significantly increased inflammation and loss of normal alveolar structures in the sections from mice depleted of pulmonary macrophages (Figs **6H, 6I, and S5C**).

### SA induced necroptosis affects multiple cell types

**Fig 7:**
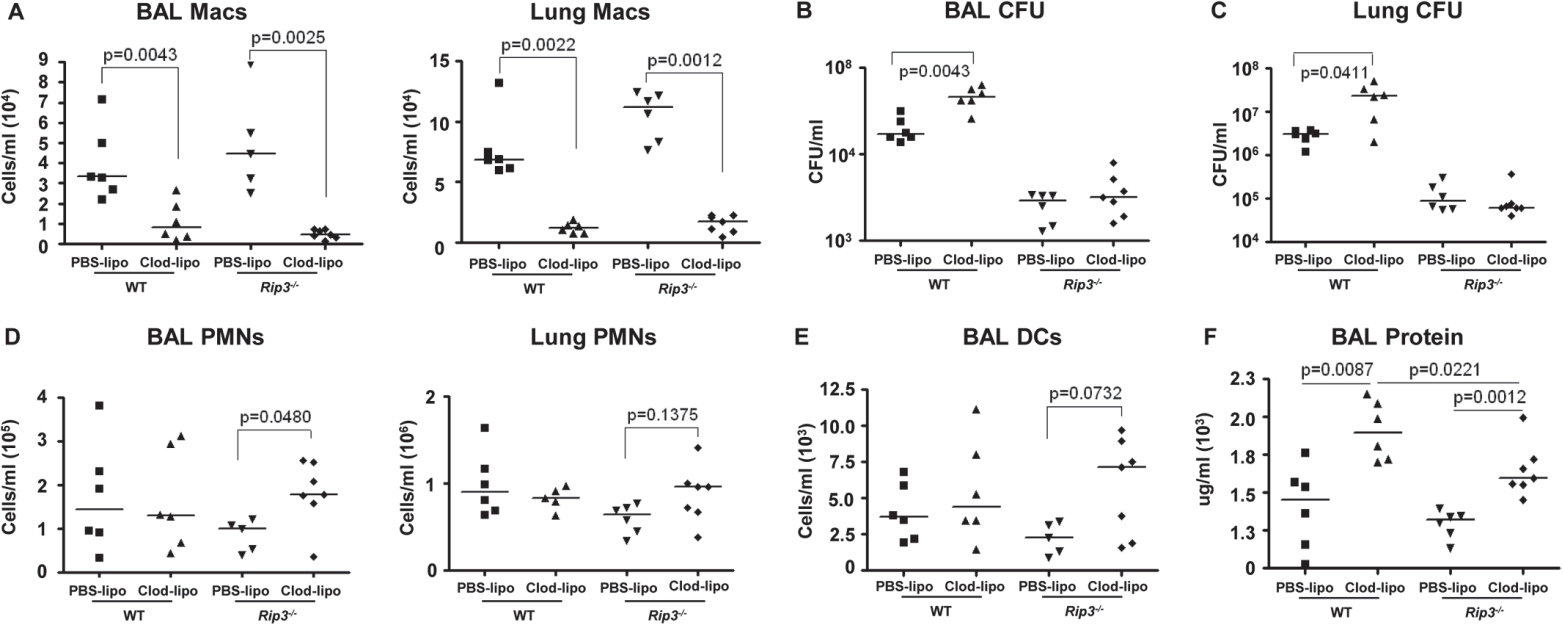
SA-induced necroptosis affects multiple cell types. (**A**) C57BL/6J and *Rip3-/-* mice were treated with clodronate- or PBS-loaded liposomes for 24 hours. Mice were infected intranasally with SA and numbers of macrophages in the BAL quantified 24 hours post-infection (n = 6 per WT and *Rip3-/-* group). (**B, C**) CFU recovered in BAL and lung of macrophage-depleted and SA-infected WT and *Rip3-/-* mice. (**D**) Numbers of PMNs in the BAL quantified 24 hours post-infection. (**E**) DCs in the BAL of macrophage-depleted and SA-infected WT and *Rip3-/-* mice. (**F**) Protein in the BAL fluid of macrophage-depleted and SA-infected WT and *Rip3-/-* mice. Data are pooled from three independent experiments. Each point in represents a mouse. Lines show median values. *p* values obtained by nonparametric Mann-Whitney test.

We next addressed whether the retention of the macrophage population in the *Rip3* ^*-/-*^ mice was solely responsible for their improved SA clearance. Given the participation of multiple staphylococcal toxins, each with specific receptors, it seemed likely that multiple immune cells would be protected by lack of *Rip3*. We evaluated outcome in *Rip3* ^*-/-*^ mice treated with PBS or clodronate loaded liposomes to deplete the macrophage population (**Fig 7A**). SA clearance was again significantly impaired in mice lacking macrophages, and *Rip3* ^*-/-*^ mice had improved clearance as compared with WT mice from both BAL and lung (**Fig 7B and 7C**). However, the depletion of macrophages from the *Rip3* ^*-/-*^ mice neither improved nor impeded their already enhanced clearance of infection (**Fig 7B and 7C**). Although macrophage depletion was comparable in the WT and *Rip3* ^*-/-*^ mice (**Fig 7A**), there was a compensatory increase in the neutrophil population in the *Rip3* ^*-/-*^ mice (**Fig 7D**), as well as a trend toward increased DCs in the lung and BAL (**Fig 7E**) making it difficult to directly compare outcomes in the two groups. Significantly greater epithelial injury was noted in the clodronate-treated mice as measured by BAL protein (**Fig 7F**). Clodronate-treated mice had greater proinflammatory cytokine expression and this phenotype was less pronounced in *Rip3* ^*-/-*^ mice (**S6A Fig**). These experiments confirm the importance of macrophage necroptosis in contributing to damaging inflammation induced by SA as well as indicating that necroptosis of other cell types targeted by staphylococcal toxins contributes to lung pathology.

## Discussion

Multiple virulence factors contribute to the pathogenesis of the severe, necrotizing pneumonia associated with MRSA USA300 [39]. The congruence and subsequent maintenance of several genetic elements in the MRSA USA300 background has enabled this strain to flourish as a human pathogen [4]. Among these virulence factors are multiple toxins, several of which contribute to host damage through the induction of cell death in host tissues. This process targets both stromal and especially immune cells, likely through the selective affinity of specific toxins for discrete leukocyte receptors resulting in the depletion of critical immune cell populations in the lung. The loss of alveolar macrophages through necroptosis and their immunoregulatory functions serve to amplify the pathological consequences of infection. The relatively increased expression of Hla and LukAB [40] further contributes to the virulence of the USA300 strain.

We focused on the effects of Hla in stimulating necroptosis, as this toxin is not species specific, enabling us to compare in vivo results from a murine model and in vitro results with human cells. Hla targets ADAM-10 resulting in E-cadherin disruption and invasive infection in the lung [5]. Our studies demonstrate that Hla also targets immune cells by specifically inducing necroptosis as evidenced by its ability to induce MLKL phosphorylation and dose-dependent induction of macrophage death that was prevented by inhibiton of MLKL. The likely mechanism responsible for toxin-mediated necroptosis involves the generation of cytoplasmic pores, as cell death was inhibited by high extracellular K^+^ or dextran, was not induced by an *hla* mutant with defective pore forming ability, and could be complemented by WT Hla, but not HlaH35L with a defective pore. Hla is a stimulus for assembly of the NLRP3 inflammasome suggesting shared signaling components for necroptosis and pyroptosis, the mode of cell death associated with inflammasome activation [6]. However, in contrast to our results obtained with the *Rip3* ^*-/-*^ mice, *Nlrp3* ^*-/-*^ mice did not have a strong phenotype in the setting of *S*. *aureus* pneumonia [10]. It is possible that stromal cells may also undergo necroptosis, thus Hla could target multiple cell types as opposed to the toxins that target only immune cells. Exactly how SA toxin pore formation leads to activation of the necrosome complex, RIP1/RIP3 and MLKL, and the shared elements through which this complex activates the inflammasome are currently under investigation. Inhibition of MLKL decreased inflammasome activation, as did lack of *Rip3* during SA infection. Although RIP3 and MLKL are reported to activate the inflammasome through ROS production [31, 41] this was not observed in the setting of SA infection, suggesting the participation of other signaling mechanisms.

Additional toxins activate necroptosis. Pore formation by both LukAB and the PSMs have been well characterized [7, 42, 43], leading to loss of plasma membrane integrity. The PSMs are α-helical peptides with varying degrees of amphipathicity enabling them to form pores in host membranes and trigger lysis [44]. Much of the cytotoxicity of these peptides is attributed to their insertion into target membranes [43]. The inability of the triple *psm* mutant studied here to activate cell death likely reflects PSMα induced membrane damage. However, the *psmα* locus is involved in Hla expression, and it is possible that the phenotype associated with the *psm* mutant used in our studies is due to decreased Hla expression [45]. LukAB is a two-component toxin that forms β-barrel pores in the host membrane [46], a mechanism of cytotoxicity that could be negated in the presence of the osmoprotectants, such as the necroptosis inhibitors used in our studies. The receptor for LukAB is human CD11b [7], which could explain the susceptibility of the human alveolar macrophage population to death induced by this toxin, but would not be expected to contribute to the cytotoxicity observed in the murine studies.

The biological significance of toxin-induced necroptosis in the pathogenesis of severe necrotizing pneumonia characteristic of USA300 infection was well illustrated in the substantial protection afforded *Rip3* ^*-/-*^ mice, which are protected from necroptosis. While multiple cell types recruited into the airway in response to infection including neutrophils are susceptible to necroptosis [47], the ability of the host to replenish most of these cells counteracts necroptotic depletion. Macrophages comprise the majority of immune cells populating the uninfected airway and these cells increase in number over the first 4 hours of infection. However, by 24 hours post-inoculation both the proportion and absolute number of macrophages decrease, a process that is significantly diminished in the *Rip3* ^*-/-*^ mice. The lost cells express the CD200R and CD206 receptors indicating their participation in anti-inflammatory signaling is critical in regulating the inflammatory milieu over the first 24 hours of infection. The *Rip3* ^*-/-*^ mice had more efficient clearance of SA from the lung, despite significantly decreased expression of KC, TNF, IL-6, IL-1α and especially IL-1β. As there were apparently sufficient numbers of neutrophils in these mice to eradicate staphylococci, there is clearly substantial redundancy in these cytokines to provide for sufficient proinflammatory activity. Thus, in contrast to published results obtained in *Il1r* ^*-/-*^ mice [48], which have a substantial defect in neutrophil recruitment, our model of infection can adequately respond to proinflammatory cytokines to generate sufficient phagocytic capabilities. The relatively decreased components of the IL-1 family appear to be beneficial in the *Rip3* ^*-/-*^ lung. While additional types of immune cells are also likely to be destroyed through necroptosis, previous studies with mice lacking DCs [49] or CD4^+^ T cells [50] indicated that these cells were not critical for bacterial clearance in the first 24 hours of infection, in contrast to results with macrophage depletion shown here. Thus, in the presence of acute infection, the loss of anti-inflammatory macrophages through toxin-induced necroptosis has important pathophysiological consequences.

The virulence of *S*. *aureus* and especially the MRSA USA300 strains has been attributed to multiple potent virulence factors that cause lung damage, elicit excessive inflammation and contribute to systemic dissemination [51]. The data presented suggest that the activation of necroptosis, a highly proinflammatory form of cell death, is a shared consequence of staphylococcal toxins. The susceptibility of specific immune cells in the lung to individual toxins and hence to necroptosis further amplifies their destructive effects in vivo. The signaling components of the necroptosis cascade may provide a final common pathway that could be targeted to prevent *S*. *aureus* induced lung damage.

## Materials and Methods

### Mice

C57BL/6J mice were sourced from Jackson Laboratory and *Rip3* ^*-/-*^ mice from Genentech [52]. Six-week-old sex-matched *Rip3* ^*-/-*^ or C57BL/6J were anesthetized with 100 mg/kg ketamine (Henry Schein) and 5 mg/kg xylazine (Henry Schein) before intranasal inoculation with 10^7^ CFU/mouse of WT MRSA or *agr* null mutant in 50 μl of PBS. To obtain the desired dilution, SA was grown to exponential phase (OD 1.000), spun down at maximum speed and resuspended in PBS. 18 to 20 hours after infection, mice were sacrificed. Bronchoalveolar lavage fluid (BAL) was obtained and lungs harvested as described below. Bacteria (CFU) were enumerated by serial dilutions on Luria Bertani (LB) agar plates and CHROMagar (Becton Dickinson) *S*. *aureus* plates at 37°C as previously described [12, 15]. Cells were stained as described below and BAL protein content measured by Bradford assay [12]. Histology was done as previously described [49].

C57BL/6J mice were treated with 300 μg RIP1 Inhibitor II, 7-Cl-O-Nec-1 (Nec-1s, Calbiochem) or DMSO 18 hours before and at the time of infection. Nec-1s- and DMSO-treated mice were infected with 10^7^ CFU/mouse MRSA USA300 and sacrificed 4 hours after infection. Bacteria were enumerated and cell stained as described above.

### Flow cytometry

Bronchoalveolar lavage fluids (BAL) were obtained by instilling 3 × 1 ml PBS into the trachea of euthanized mice before lungs were harvested. Lungs were obtained and passed through a cell strainer (Fisher Scientific) with 400 μl PBS to obtain a single-cell homogenate. BAL fluid was centrifuged at 200x *g* for 10 minutes and lung homogenate at 400x *g* for 6 minutes. For flow cytometry analysis, red blood cells were lysed and remaining cells washed with FACS buffer. Cells were suspended in 100 μl FACS Buffer (10% fetal bovine serum and 0.1% sodium azide in PBS) and stained for 30 minutes at 4°C in presence of counting beads (Bang Laboratories, Inc.). Combinations of fluorescein isothiocyanate-labelled (FITC) anti-Ly-6G (Gr-1; RB6-8C5; Biolegend), peridinin chlorophyll (PerCP)-Cy5.5-labelled anti-CD11c (N418; Biolegend), allophycocyanin (APC)-labelled anti-MHC II (I-A/I-E; Biolegend), phycoerythrin(PE)-labelled anti-NK 1.1 (NKR-P1C, Ly-55; Biolegend), FITC-CD200R (Biolegend), PE-CD86 (eBiosciences), FITC-CD54 (Biolegend), PE-CD206 (Biolegend) and propidium iodide (PI, Life Technologies) were used. Neutrophils (Ly6G^+^/MHCII^−^), macrophages (CD11C^+^/MHCII^low-mid^) and dendritic cells (CD11c^+^/MHCII^high^) were analyzed using WinMIDI Version 2.9 and FlowJo vX.0.7.

### Lung macrophage depletion

Lung macrophages were depleted by intranasally administering 75 μl clodronate liposomes or PBS liposomes (ClodronateLiposomes.org, Netherlands). Mice were infected and sacrificed 4 and 24 hours later. Lung and BAL were analyzed as previously described [13]. Macrophage depletion was confirmed by flow cytometry in BAL and lung samples.

### Bacterial strains

Wild type MRSA USA300 FPR3757, MRSA USA300 LAC and their mutants were grown overnight in LB Broth at 37°C to stationary phase. *S*. *aureus* were diluted to 1:100 in LB and grown to OD 1.000 and resuspended in PBS to achieve the required density. Heat-killed SA was obtained by incubating SA cultures at OD1.000 at 65°C for 1.5 h to inactivate the bacteria. USA300 LAC (WT), α-hemolysin (*hla*), *pha*H35L and the complemented *phla* mutants were obtained from Juliane Bubeck-Wardenburg (University of Chicago, IL); USA300 LAC WT and Panton-Valentine leukocidin (*pvl; lukSF*) mutant from Frank DeLeo (National Institute of Allergy and Infectious Diseases, Rocky Mountain Laboratories, MT); α, β and γ phenol-soluble modulins (*PSMα/β/hld*) triple mutant from Michael Otto (National Institute of Allergy and Infectious Diseases, MD); and *lukAB* from Victor Torres (NYU Langone Medical Center, NY). SA supernatant was obtained by growing WT MRSA USA300 and mutant strains overnight to stationary phase in LB. Cells were spun at high speed and supernatant passed through a sterile filter with 20 μm pore size (Millipore).

### Cell culture

THP-1 human monocytic cells (THP-1 cells, ATCC) were grown in RPMI1640 medium (CellGro) with 10% fetal bovine serum (FBS, Mediatech) and 1% penicillin/streptomycin solution (Life Technologies) at 37°C, 5% CO_2_ in a humidified environment. THP-1 cells were activated with 1 μM PMA (Sigma-Aldrich) for 24 hours and weaned to RPMI1640 medium without antibiotics for 24 hours. Human primary macrophages were isolated from 40 ml of heparinized blood from consenting, healthy anonymous volunteers. Human macrophages were grown for 7 days in RPMI 1640 medium supplemented with 10% fetal bovine serum (FBS), penicillin-streptomycin and 60 ng/mL M-CSF (PeproTech). Human primary macrophages or THP-1 cells were pretreated 1 hour before infection with stated concentrations of necrostatin-1 (Nec-1, Enzo Life Technologies), 200 μM necrostatin-1 stable (Nec-1s, Enzo Life Technologies), 10 μM necrosulfonamide (NSA, Calbiochem), DMSO control (v/v), 10 mM N-acetyl-L-cysteine (NAC, Sigma), 1 mM or 2.5 mM 100,000 MW dextran (Sigma) or 60 mM KCl (Sigma). *S*. *aureus* mutants and their isotype controls at multiplicity of infection (MOI) 10 were added to THP-1 cells for 2 hours or stated time points and the THP-1 cells supernatant and lysates obtained.

Alveolar macrophages were isolated from de-identified human bronchoalveolar lavage (BAL) fluid that would otherwise be discarded (Columbia University IRB-AAAI5300). BAL was run through a sterile cell strainer (Fisher Scientific) to remove mucus, treated with RBC lysis buffer, washed in PBS and resuspended in 1640 RPMI medium supplemented with 10% FBS. Cells were pretreated with 10 μM necrosulfonamide (NSA) or DMSO (v/v) for 1 hour and stimulated with MRSA USA300 MOI100 for 2 hours. Cytotoxicity was measured by lactate dehydrogenase (LDH) assay (Roche).

### Cytotoxicity assays

LDH assay was performed as per manufacturer’s instructions (Roche). Multiply frozen-thawed THP-1 cells were used as a maximal LDH release or PI (Life Technologies) staining (100%) in RPMI 1640 medium and the medium alone used as a baseline. Its fluorescence intensity was read on a Tecan Microplate Reader (Tecan Group). THP-1 cells were stained with PI (in experiments with NAC, which reacts with LDH reagents) and analyzed using Tecan Microplate Reader.

### siRNA knockdown

THP-1 cells grown in 24-well plates to confluence were transfected with 100 nM RIP3, non-targeting siRNA (human ONTARGETplus siRNA pools of four oligos; Dharmacon) or MLKL (human MLKL siRNA pools of three targets, Santa Cruz Biotechnology) using Lipofectamine RNAiMAX (Life Technologies). After 72 hours, cells were stimulated with MRSA USA300 LAC (MOI 10) or PBS for 2 h and THP-1 cells supernatant used to determine the level of cytotoxicity.

### ELISA and western blot analysis

Human IL-1β (R&D Systems) was quantified in THP-1 supernatants and mouse IL-6 (Biolegend), CXCL1/KC (R&D Systems), IL-1α (Biolegend), IL-1β (Biolegend) and TNF (eBioscience) were quantified in mouse BAL fluid by ELISA according to manufacturer’s instructions. THP-1 cells were were lysed using RIPA buffer (20 mM Tris-Cl, 50 mM NaCl, 0.1% SDS, 1% Triton X-100, 10% glycerol, 2 mM EDTA, 0.5% sodium deoxycholate) containing 1X Halt protease and phosphatase single-use inhibitor cocktail (Thermo Scientific). To probe for secreted SA toxin expression, SA cultures were grown overnight to stationary phase and their supernatant filtered. Proteins were separated on Bolt 4%–12% Bis-Tris Plus gels (Life Technologies), transferred using iBLot Dry Blotting System (Life Technologies), and blocked with 5% milk in TBST (Tris-buffered saline plus Tween) (50 mM Tris, pH 7.5, 150 mM NaCl, 0.05% Tween) or 5% bovine serum albumin (BSA) in TBST for 1 hour at room temperature. Immunodetection was performed using anti-MLKL (phospho S358) (Abcam), anti-MLKL (Abcam), RIP3 (Santa Cruz Biotechnology Inc.), anti-caspase-1 (Santa Cruz Biotechnology Inc.), β-actin (Sigma-Aldrich), Hla (a generous gift from Juliane Bubeck-Wardenburg) and LukF (IBT Bioservices) antibodies followed by secondary antibodies conjugated to horseradish peroxidase (Santa Cruz Biotechnology Inc.). 0.1% Ponceau S (w/v) in 5% acetic acid (Sigma) was used to confirm equal loading of SA supernatants. Densitometry was determined by ImageJ.

### RNA analysis

RNA isolation and analysis was performed as described previously [53]. Sequences for mouse *Tnf*, *KC*, *Il-6* and *Actin* primers have been published elsewhere [53]. Human primers for *Actin* were 5’-GTGGGGCGCCCCAGGCACCA-3’; and 5’-CGGTTGGCCTTGGGGTTCAGGGGGG-3’; *RIPK3* were 5’-CTCTCTGCGAAAGGACCAAG-3’ and 5’- CATCGTAGCCCCACTTCCTA-3’; *MLKL* were 5’-CTCTTTCCCCACCATTTGAA-3’ and 5’-TCATTCTCCAGCATGCTCAC-3’. All RNA expressions were normalized to actin.

### Ethics statement

Animal work in this study was carried out in strict accordance with the recommendations in the Guide for the Care and Use of Laboratory Animals of the National Institutes of Health, the Animal Welfare Act and U.S. federal law. The Institutional Animal Care and Use Committee (IACUC) of Columbia University approved the protocol (AAAG9307). Alveolar macrophages were isolated from de-identified human bronchoalveolar lavage fluid (Columbia University IRB-AAAI5300) that would otherwise be discarded. All subjects enrolled provided voluntary informed written consent and signed a copy of the appropriate and stamped consent documents. Copies of the consent documents were given to the subjects for their record. Human primary macrophages were isolated from 40 ml of heparinized blood from consenting, healthy anonymous volunteers with informed oral consent (Columbia University IRB-AAAC5450). Columbia University Institutional Review Board approved a waiver for documentation of consent for blood collection because blood draws involve minimal risk.

### Statistical analysis

Samples with normal distribution were analyzed by two-tailed Student's *t* test. For multiple comparisons, one-way ANOVA was used followed by Bonferroni Corrections to correct for multiple comparisons. Mouse cytokines, bacterial counts, and immune cell numbers and other samples with non-normal distributions were analyzed using the nonparametric Mann-Whitney test. Outliers were determined by Grubb’s test and removed (GraphPad Software). Differences in groups were considered significant if *p* < 0.05. Statistical analysis was determined using GraphPad Prism Version 4.00. For scatter blots, data are presented as single points with the median value represented by a bar.

## Supporting Information

S1 Fig Hla expression and level of IL-1β produced in cells stimulated with purified Hla. (**A**) Levels of IL-1β as quantified by ELISA in THP-1 cells pretreated with 10 μM NSA or DMSO and exposed to purified Hla for 2 hours (*p < 0.05). (B) Hla expression in supernatant from *hla* mutants as assayed by western blot. Data are representative of two independent experiments with. *p* values were determined by two-tailed Student's *t* test (**B**).
(TIF)

S2 Fig Immune response to SA in *Rip3* ^*-/-*^ and Nec-1s-treated mice. C57BL/6J mice were treated with necrostatin-1 stable (Nec-1s) or DMSO and infected with MRSA USA300 (SA) (n = 2 for PBS and n = 10 for SA group). (**A**) CXCL1/KC, IL-6, TNF and IL-1β levels in the BAL fluid measured by ELISA. (**B**) Natural killer cells (NKs) in BAL and lung and CD4+ T cells (CD4s) in lung in Nec-1s-treated mice. (**C**) *Rip3* ^*-/-*^ or wild type C57BL/6J (WT) mice were infected with SA for 18 hours. CXCL1/KC, IL-6, TNF and IL-1β levels in lung as measured by quantitative RT PCR (mean, SD) (n = 3 for PBS and n = 8 for SA group). (**D**) H&E staining of *Rip3* ^*-/-*^ or WT mice infected with *agr* null or MRSA USA300 (USA300) for 18 hours (magnification of 100x; insert, magnification of 400x). (**E**) FACS blots showing gating strategies for immune cells. Data are pooled from two independent experiments. Each point represents a mouse. Lines show mean or median values (**A, B**). *p* values were determined by nonparametric Mann-Whitney test.
(TIF)

S3 Fig Immune cells expansion during SA pneumonia. (**A-E**) Mice were infected intranasally with 10^7^ CFU/mouse SA and their lung homogenate analyzed for immune cell populations at 4 and 24 hours as compared to uninfected controls (UN) (n = 9 for UN, n = 9 for 4 h and n = 20 for 24 h SA groups). (**A, B**) DCs in BAL and lung quantified 4 and 24 hours after SA infection. (**C**) NKs in lung quantified 4 and 24 hours after SA infection. (**D**) CD4^+^ T cells in lung quantified 4 and 24 hours after SA infection. (**E**) FACS blots showing propidium iodide positive (PI^+^) macrophages in the BAL of WT and *Rip3* ^*-/-*^ mice (n = 3 for PBS and n = 8 per SA group). (**F**) CD4^+^ T cells in lung of WT and *Rip3* ^*-/-*^ mice. (**G**) DCs in BAL and lung of WT and *Rip3* ^*-/-*^ mice. (**H**) DCs in BAL and lung of WT and *Rip3* ^*-/-*^ mice. Data are pooled from three independent experiments. Each point in represents a mouse. Lines show median values. *p* values are indicated for significantly different comparisons (nonparametric Mann-Whitney test).
(TIF)

S4 Fig Pulmonary macrophages from *Rip3* ^*-/-*^ mice have an anti-inflammatory phenotype. (**A**) *Rip3* ^*-/-*^ or WT mice were infected with SA for 18 hours and percentages of CD54^+^ macrophages in BAL and lung quantified (n = 3 for PBS, n = 8 for SA groups). (**B**) CD86^+^ macrophages in BAL and lung (n = 4 for PBS, n = 6 for WT with SA, n = 7 for *Rip3* ^*-/-*^ with SA). (**C**) CD206^+^ macrophages in BAL and lung (n = 3 for PBS, n = 7 for WT with SA, n = 8 for *Rip3* ^*-/-*^ with SA). (**D**) CD200 receptor positive (CD200R^+^) macrophages in BAL and lung (n = 3 for PBS, n = 7 for WT with SA, n = 8 for *Rip3* ^*-/-*^ with SA). (**E**) FACS blots showing gating strategy for macrophage markers. Data are pooled from two independent experiments. Each point represents a mouse. Lines show median values. *p* values were determined by nonparametric Mann-Whitney test.
(TIF)

S5 Fig Effects of depletion of pulmonary macrophages in SA pneumonia. (**A**) C57BL/6J mice were treated with clodronate- or PBS-loaded liposomes for 24 hours. Mice were infected intranasally with SA and DCs in BAL and lung was quantified 4 hours and 24 hours post-infection by flow cytometry (n = 4 for PBS groups, n = 10 for SA groups). (**B**) CD4^+^ T cells in lung 4 hours and 24 hours post-infection. (**C**) Hematoxylin and eosin stain (H&E) staining of mouse lung (magnification 100x; insert, magnification 400x). (**D**) FACS blots showing depleted macrophages and unchanged PMNs after clodronate treatment. Data are pooled from three independent experiments. Each point in represents a mouse. Lines show median values. *p* values obtained by nonparametric Mann-Whitney test.
(TIF)

S6 Fig Cytokines in macrophage-depleted *Rip3* ^*-/-*^ mice. (**A**) C57BL/6J and *Rip3* ^*-/-*^ mice were treated with clodronate- or PBS-loaded liposomes for 24 hours (n = 6 per WT and *Rip3* ^*-/-*^ group). Mice were infected intranasally with SA and lCXCL1/KC, IL-6, TNF and IL-1β levels in the BAL fluid measured by ELISA. Data are pooled from three independent experiments. Each point in represents a mouse. Lines show median values. *p* values obtained by nonparametric Mann-Whitney test.
(TIF)
