# Supplementary figures and images for: Toxin-Induced Necroptosis Is a Major Mechanism of Staphylococcus aureus Lung Damage
Source: PLoS Pathog. 2015 Apr 16;11(4):e1004820. doi: 10.1371/journal.ppat.1004820 (PMC4399879; doi:10.1371/journal.ppat.1004820)

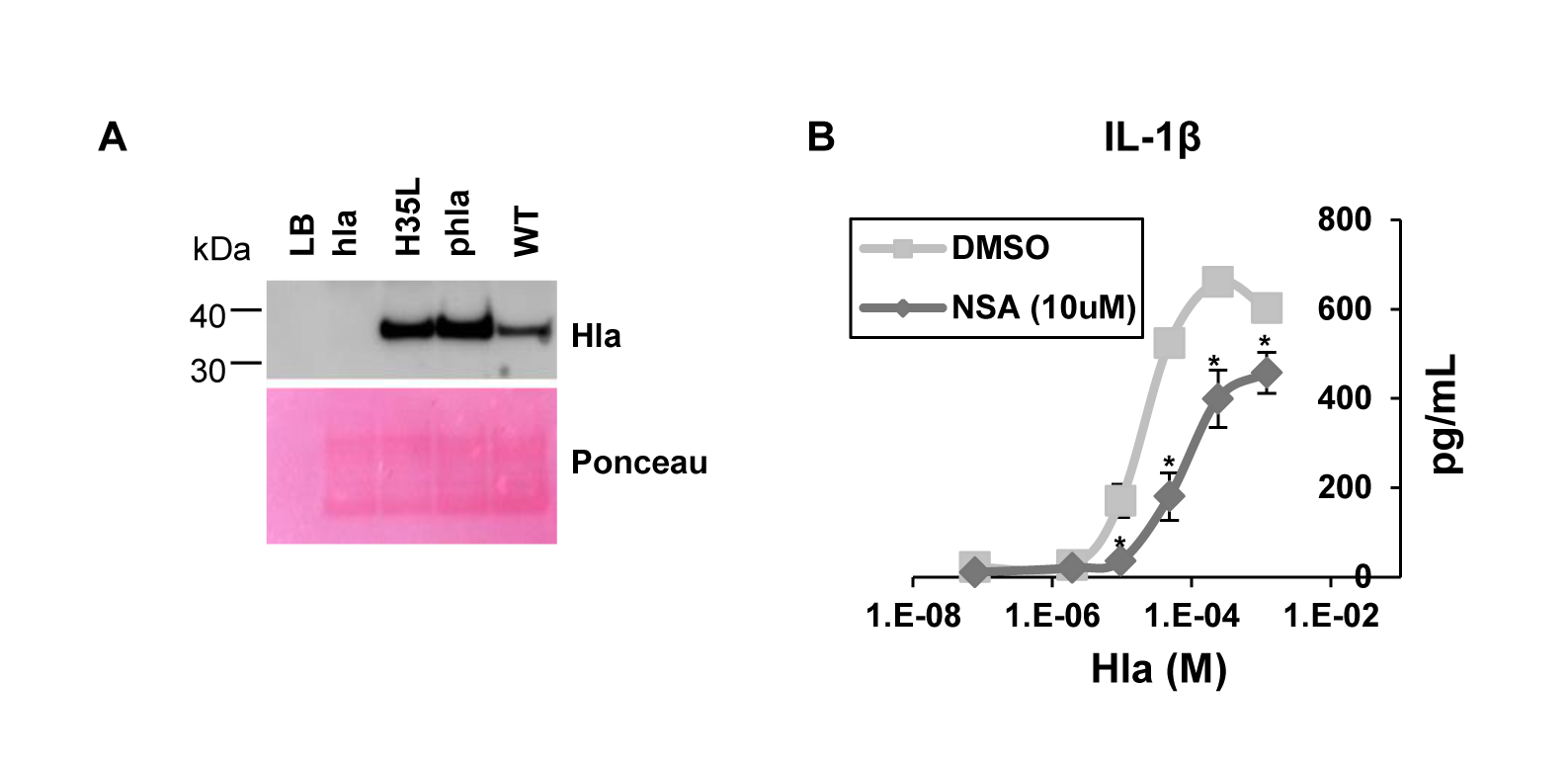

Supplement: S1 Fig — (A) Levels of IL-1β as quantified by ELISA in THP-1 cells pretreated with 10 μM NSA or DMSO and exposed to purified Hla for 2 hours (*p < 0.05). (B) Hla expression in supernatant from hla mutants as assayed by western blot. Data are representative of two independent experiments with. p values were determined by two-tailed Student's t test (B). (TIF) [file ppat.1004820.s001.tif]

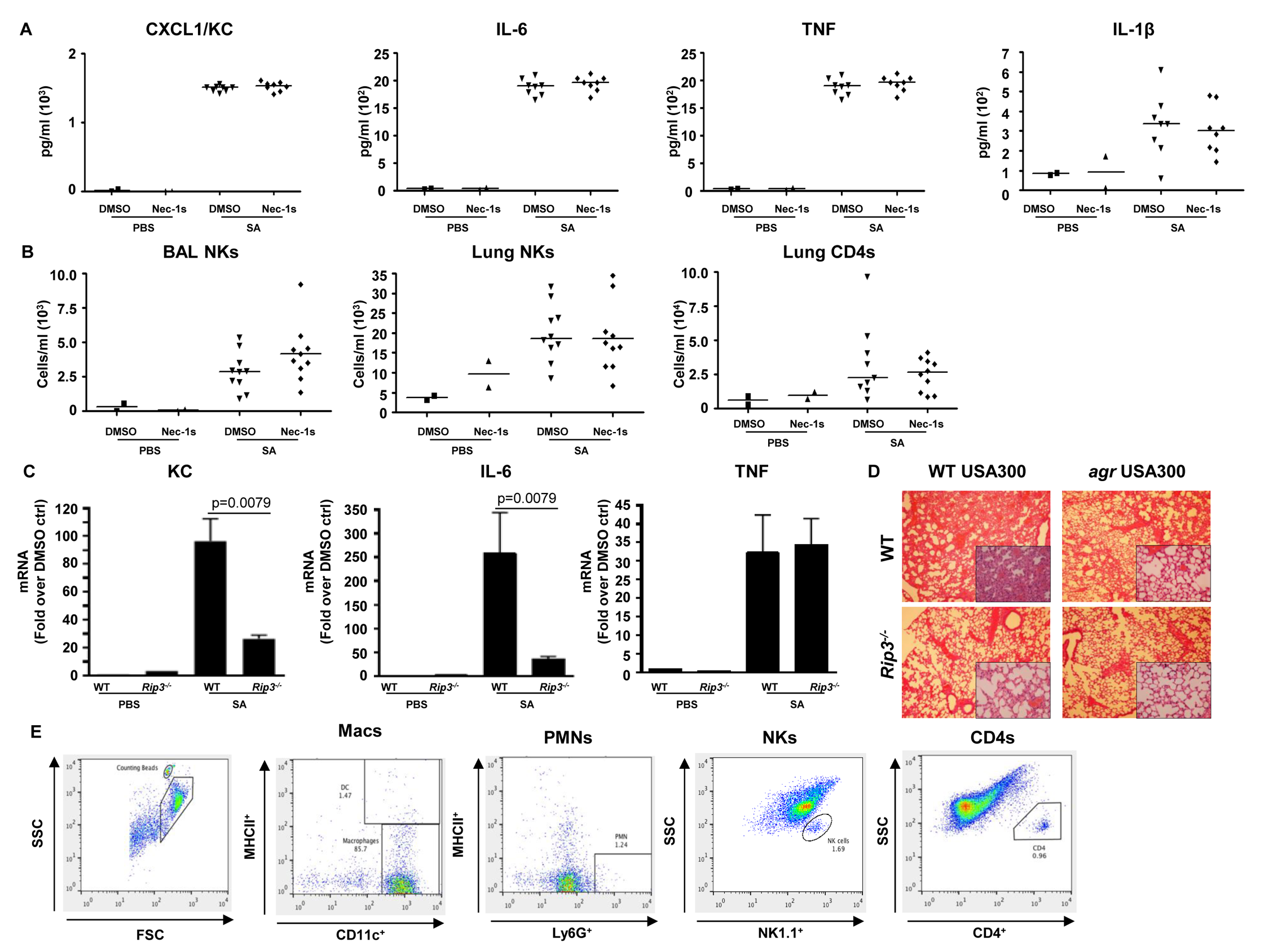

Supplement: S2 Fig — C57BL/6J mice were treated with necrostatin-1 stable (Nec-1s) or DMSO and infected with MRSA USA300 (SA) (n = 2 for PBS and n = 10 for SA group). (A) CXCL1/KC, IL-6, TNF and IL-1β levels in the BAL fluid measured by ELISA. (B) Natural killer cells (NKs) in BAL and lung and CD4+ T cells (CD4s) in lung in Nec-1s-treated mice. (C) Rip3 -/- or wild type C57BL/6J (WT) mice were infected with SA for 18 hours. CXCL1/KC, IL-6, TNF and IL-1β levels in lung as measured by quantitative RT PCR (mean, SD) (n = 3 for PBS and n = 8 for SA group). (D) H&E staining of Rip3 -/- or WT mice infected with agr null or MRSA USA300 (USA300) for 18 hours (magnification of 100x; insert, magnification of 400x). (E) FACS blots showing gating strategies for immune cells. Data are pooled from two independent experiments. Each point represents a mouse. Lines show mean or median values (A, B). p values were determined by nonparametric Mann-Whitney test. (TIF) [file ppat.1004820.s002.tif]

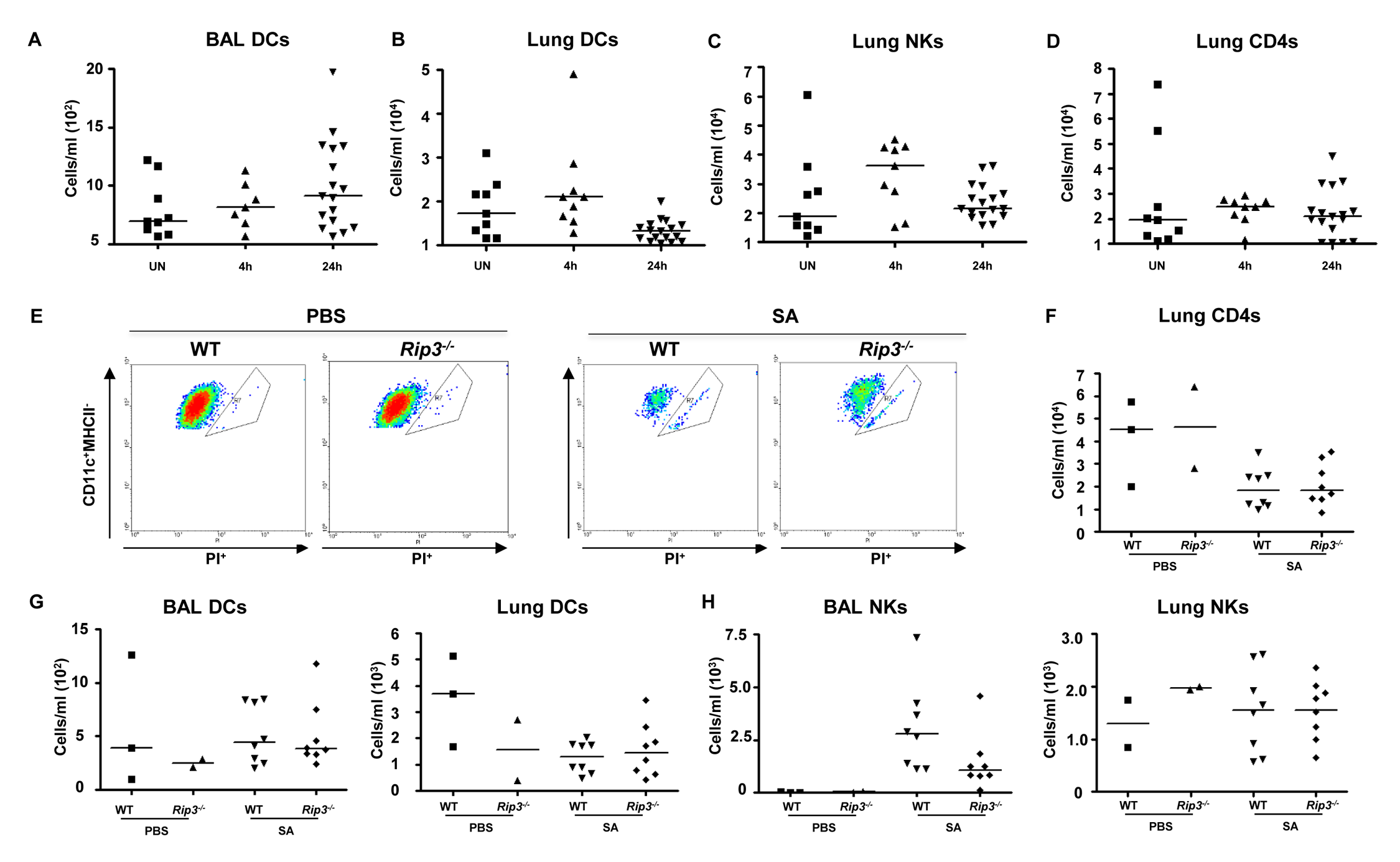

Supplement: S3 Fig — (A-E) Mice were infected intranasally with 107 CFU/mouse SA and their lung homogenate analyzed for immune cell populations at 4 and 24 hours as compared to uninfected controls (UN) (n = 9 for UN, n = 9 for 4 h and n = 20 for 24 h SA groups). (A, B) DCs in BAL and lung quantified 4 and 24 hours after SA infection. (C) NKs in lung quantified 4 and 24 hours after SA infection. (D) CD4+ T cells in lung quantified 4 and 24 hours after SA infection. (E) FACS blots showing propidium iodide positive (PI+) macrophages in the BAL of WT and Rip3 -/- mice (n = 3 for PBS and n = 8 per SA group). (F) CD4+ T cells in lung of WT and Rip3 -/- mice. (G) DCs in BAL and lung of WT and Rip3 -/- mice. (H) DCs in BAL and lung of WT and Rip3 -/- mice. Data are pooled from three independent experiments. Each point in represents a mouse. Lines show median values. p values are indicated for significantly different comparisons (nonparametric Mann-Whitney test). (TIF) [file ppat.1004820.s003.tif]

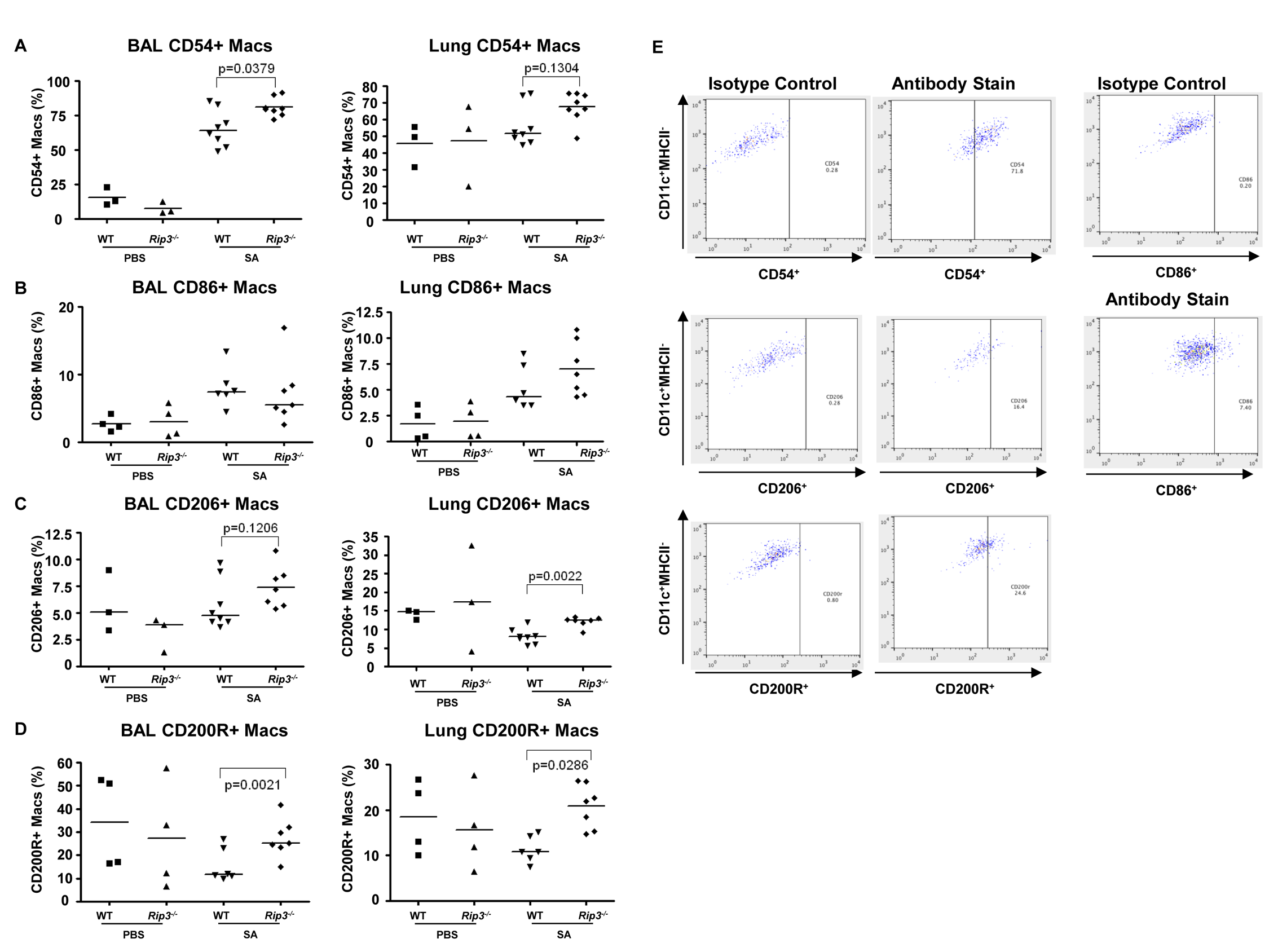

Supplement: S4 Fig — (A) Rip3 -/- or WT mice were infected with SA for 18 hours and percentages of CD54+ macrophages in BAL and lung quantified (n = 3 for PBS, n = 8 for SA groups). (B) CD86+ macrophages in BAL and lung (n = 4 for PBS, n = 6 for WT with SA, n = 7 for Rip3 -/- with SA). (C) CD206+ macrophages in BAL and lung (n = 3 for PBS, n = 7 for WT with SA, n = 8 for Rip3 -/- with SA). (D) CD200 receptor positive (CD200R+) macrophages in BAL and lung (n = 3 for PBS, n = 7 for WT with SA, n = 8 for Rip3 -/- with SA). (E) FACS blots showing gating strategy for macrophage markers. Data are pooled from two independent experiments. Each point represents a mouse. Lines show median values. p values were determined by nonparametric Mann-Whitney test. (TIF) [file ppat.1004820.s004.tif]

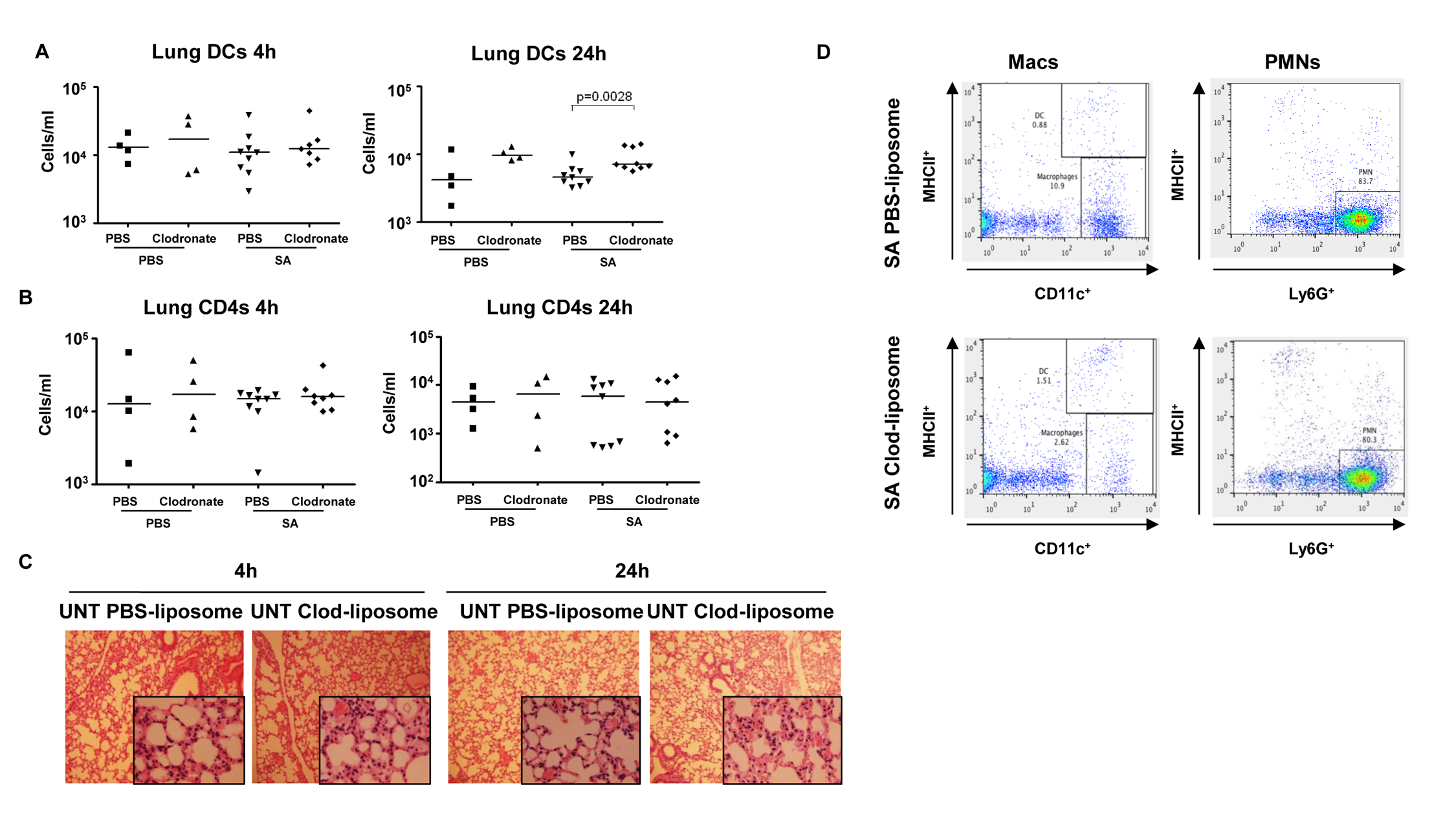

Supplement: S5 Fig — (A) C57BL/6J mice were treated with clodronate- or PBS-loaded liposomes for 24 hours. Mice were infected intranasally with SA and DCs in BAL and lung was quantified 4 hours and 24 hours post-infection by flow cytometry (n = 4 for PBS groups, n = 10 for SA groups). (B) CD4+ T cells in lung 4 hours and 24 hours post-infection. (C) Hematoxylin and eosin stain (H&E) staining of mouse lung (magnification 100x; insert, magnification 400x). (D) FACS blots showing depleted macrophages and unchanged PMNs after clodronate treatment. Data are pooled from three independent experiments. Each point in represents a mouse. Lines show median values. p values obtained by nonparametric Mann-Whitney test. (TIF) [file ppat.1004820.s005.tif]

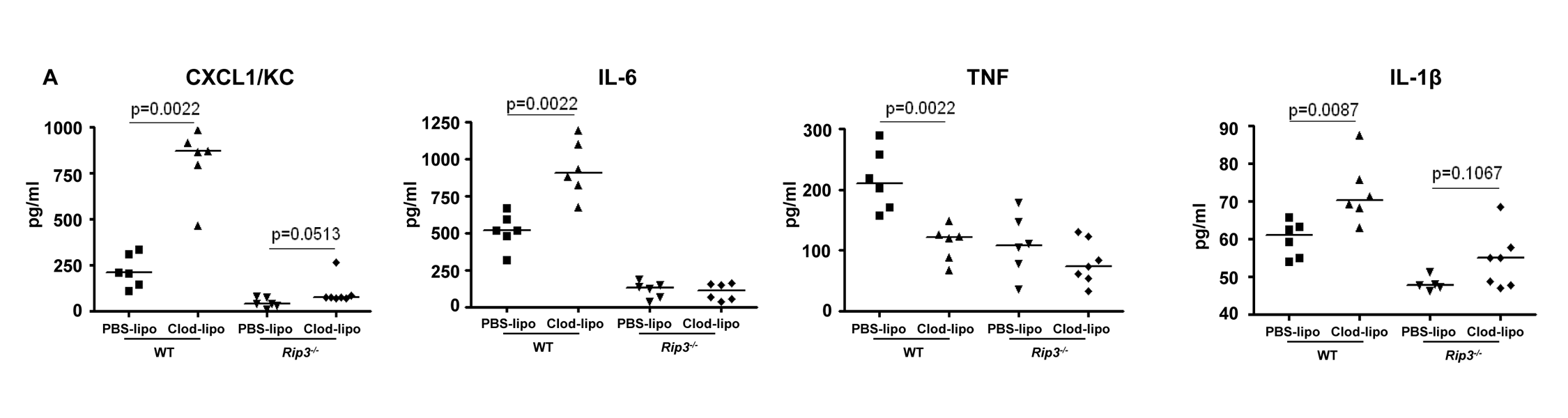

Supplement: S6 Fig — (A) C57BL/6J and Rip3 -/- mice were treated with clodronate- or PBS-loaded liposomes for 24 hours (n = 6 per WT and Rip3 -/- group). Mice were infected intranasally with SA and lCXCL1/KC, IL-6, TNF and IL-1β levels in the BAL fluid measured by ELISA. Data are pooled from three independent experiments. Each point in represents a mouse. Lines show median values. p values obtained by nonparametric Mann-Whitney test. (TIF) [file ppat.1004820.s006.tif]
